# Acute responses of regional vascular conductance to oral ingestion of fructose in healthy young humans

**DOI:** 10.1186/1880-6805-33-11

**Published:** 2014-05-17

**Authors:** Masako Y Endo, Chizuko Fujihara, Chinami Yamazaki, Hideaki Kashima, Kouhei Eguchi, Akira Miura, Yoshiyuki Fukuoka, Yoshiyuki Fukuba

**Affiliations:** 1 Department of Exercise Science and Physiology, School of Health Sciences, Prefectural University of Hiroshima, 1-1-71 Ujina-higashi, Minami-ku, Hiroshima, 734-8558, Japan; 2 Faculty of Health and Sports Science, Doshisha University, Kyotanabe, Kyoto, 610-0394, Japan

**Keywords:** fructose, blood pressure, vascular conductance, central circulation, peripheral circulation

## Abstract

**Background:**

Recently, it was reported in healthy young subjects that fructose containing drinks increased blood pressure acutely, without any apparent change in total vascular conductance (TVC). However, because it is well known that the splanchnic vasculature is dilated by oral fructose ingestion, it is assumed to be the concomitant vasoconstriction in other peripheral region(s) that is responsible for this finding. Thus, the purpose of this study was to determine the acute response of regional VC to oral fructose ingestion in young healthy humans.

**Results:**

In 12 healthy young subjects, mean arterial blood pressure (MAP), heart rate, cardiac output, and blood flow (BF) in the superior mesenteric (SMA), brachial (BA), and popliteal (PA) arteries, in addition to forearm skin BF, were measured continuously for 2 h after ingestion of 400 ml fructose solution (containing 50 g fructose). Regional VC was calculated as BF/MAP. MAP increased for 120 min after fructose ingestion without any change in TVC. While VC in the SMA was elevated after ingestion, VC in BA and PA and forearm skin decreased.

**Conclusions:**

While TVC was apparently unchanged during the 2 h after fructose ingestion, there were coincident changes in regional VCs in the peripheral circulation, but no net change in TVC.

## Background

In recent decades, daily consumption of dietary fructose has increased gradually. In addition to fruit, carbonated beverages containing a form of high-fructose corn syrup have been implicated in the increase [1]. Long-term consumption of fructose results in an increase in plasma triglycerides in humans and animals [2-6], and is considered a significant dietary problem in advanced countries associated with obesity and arteriosclerosis [7-9]. Several recent studies have suggested that dietary fructose consumption may lead to the development of cardiovascular diseases such as hypertension [5,8,10,11]. The current understanding to the background of fructose-derived hypertension is unclear as to whether overconsumption of fructose itself induced the elevation of BP directly or indirectly (via hyperuricemia, which is subsequently followed by activation of the rennin-angiotensin-aldosterone system and damage to the renal tubule [11,12]).

The blood pressure (BP) was greater after consumption of a high fructose diet for eight weeks compared with rats consuming a normal diet in rats [11]. In older people, the BP acutely increased following ingestion of fructose-containing drinks [13]. Even in healthy young subjects, Brown *et al*. [7] showed that oral consumption of a 50-g pure fructose-containing drink rapidly increased BP, which was sustained for at least a few hours, whereas there was no comparable effect after consumption of either a glucose-containing drink or water [7]. They suggested that fructose-specific acute elevations in BP were mainly mediated not by a decreased peripheral vascular response (that is, no change in total vascular conductance, TVC), but by a sustained elevation of cardiac output (CO). However, it is well known that fructose ingestion results in a slight, but significant, increase in acute splanchnic circulation [14]. Together with these results, it is hypothesized that regional VC in areas such as the viscera, limbs, and/or skin would be acutely changed, even while TVC remains apparently unchanged. Therefore, we did this study as a pilot to reconfirm the results by Brown *et al*. [7], including the simultaneous measurements of peripheral VCs in several targeted arteries with our technique (that is, pulsed Doppler ultrasound sonography).

Based on the findings above, focusing on regional VC after fructose ingestion is important because the accumulation of acute effect(s) to regional cardiovascular regulation may be a precursor to a more chronic phenomenon, that is, fructose-induced hypertension. Thus, the purpose of this study was to determine acute responses of regional VC to oral fructose ingestion in young healthy humans.

## Methods

### Subjects

Twelve healthy young subjects (four males, eight females) were studied. The mean (± SEM) age, height, and body mass of the subjects was 21.5 ± 1.4 years, 160.0 ± 6.4 cm, and 50.8 ± 4.3 kg, respectively. All subjects were informed of the purpose, protocol, and risks associated with the procedures before giving written informed consent to participate in the study, which was approved by the local Ethics Committee of Hiroshima and was conducted in accordance with the Declaration of Helsinki. The menstrual cycle in the female subjects was not controlled.

### Protocol

The subjects arrived at the laboratory in the morning after a 12-hour fast. After baseline measurements for 30 min, the subjects ingested a 400-ml fructose solution. Then, the subjects rested for 120 min in a supine position, except at the time of drink ingestion. The subjects consumed the drink within 3 min in a sitting position. The test drink was a water-based solution containing 50 g fructose. Unsweetened lemon juice (10 ml) was added to the drink to provide a more appetizing taste for the participants. The ambient temperature of the experimental room was kept at 23 ± 0.5 °C and 40-50% relative humidity by a thermal feedback device.

### Measurements

Heart rate (HR) was continuously measured using an electrocardiogram (BPM-300, Nippon Colin Co., Komaki, Japan) throughout the protocol. Measurement of mean arterial pressure (MAP) was performed using the same instrument on the left arm by an automated oscillometric method every 10 min. The beat-to-beat stroke volume (SV) was estimated using the model flow method (Finometer PRO, Finapres Medical Systems, Amsterdam, The Netherlands) on the left middle finger, which provides a reliable estimate of changes in SV, even during exercise in healthy humans [15]. Cardiac output (CO) and total vascular conductance (TVC) were calculated using the following equations:

CO=SV×HRandTVC=CO/MAP,respectively.

Blood velocity (BV) and vessel diameter were obtained on a beat-by-beat basis using pulsed Doppler ultrasound sonography (LOGIQ S6, GE Medical Systems, Tokyo, Japan) on the right brachial and popliteal arteries (BA and PA) every 20 min with a 5.0 MHz linear probe, and with a 3.5-MHz convex probe on the superior mesenteric and right renal arteries (SMA and RA). The BA measurement was taken 5 cm from the axilla, and the PA measurement was taken on the popliteal fossa. The locations of SMA and RA measurements were within 1 to 3 cm from the branch of each artery. To obtain the highest quality Doppler tracings, the optimal positions of the Doppler probe for each subject were determined in a preliminary trial (that is, the proper alignment of the ultrasound beam with the artery). After adjustment of the sample volume width to cover the target arterial diameter, the Doppler probe was kept constant on the subject’s skin surface. Ultrasound insonation resulted in an angle between 45 and 60°. To avoid the effect of movement of the SMA and RA during the cyclic phase of breathing, subjects were told to hold their breath for approximately 10 s with natural expiration. The BV at each time point was averaged during approximately 10 to 20 cardiac cycles. Vessel diameter was obtained by analyzing pictures of the vertical section of blood vessels using a B-mode ultrasound. BV was calculated as:

$$\mathrm{BF}=\mathrm{BV}\times {\left(\text{vessel}\;\text{diameter}\right)}^{2}\times \pi /4.$$

The details were described in our previous studies [16,17]. These BFs were always measured in the order BA, PA, SMA, and RA within 5 min at 20-min intervals. The VC of each artery was calculated using the following equation:

VC=BF/MAP.

The BF of skin in the right forearm was measured continuously using a laser rheometer (ALF21; Advance, Tokyo, Japan).

### Statistical analysis

All data are expressed as mean ± SEM. The effect of time on the variables was examined using repeated measures one-way analysis of variance (ANOVA). When significant differences were detected, Dunnett’s post-hoc test was used to reveal the effect of time against the baseline (that is, values before ingestion). The level of statistical significance was set at P <0.05. All analyses were performed using SPSS 12.0 for Windows (SPSS, Chicago, IL, USA).

## Results and discussion

**Figure 1:**
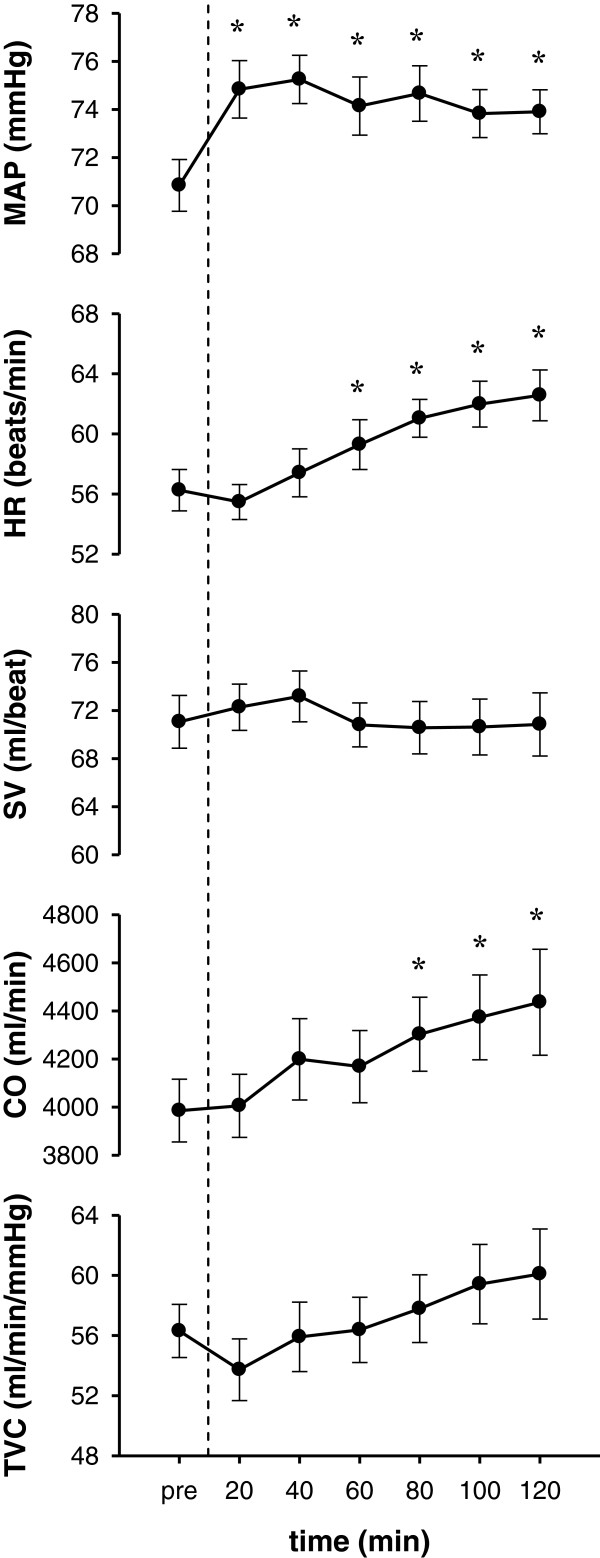
**Changes in circulation parameters before and after ingestion of fructose.** Changes in mean arterial pressure (MAP), heart rate (HR), stroke volume (SV), cardiac output (CO), and total vascular conductance (TVC) are shown from the top. Data: mean ± SEM*: versus pre-ingestion (*P* <0.05).

Changes in MAP, HR, SV, CO, and TVC after ingestion compared with baseline (that is, before ingestion) are shown in Figure 1. MAP increased significantly after ingestion and remained elevated for 120 min. HR and CO increased gradually and were significantly higher compared with baseline from approximately 60 min after ingestion. TVC did not change significantly, but there was a trend to increase gradually with time.

**Figure 2:**
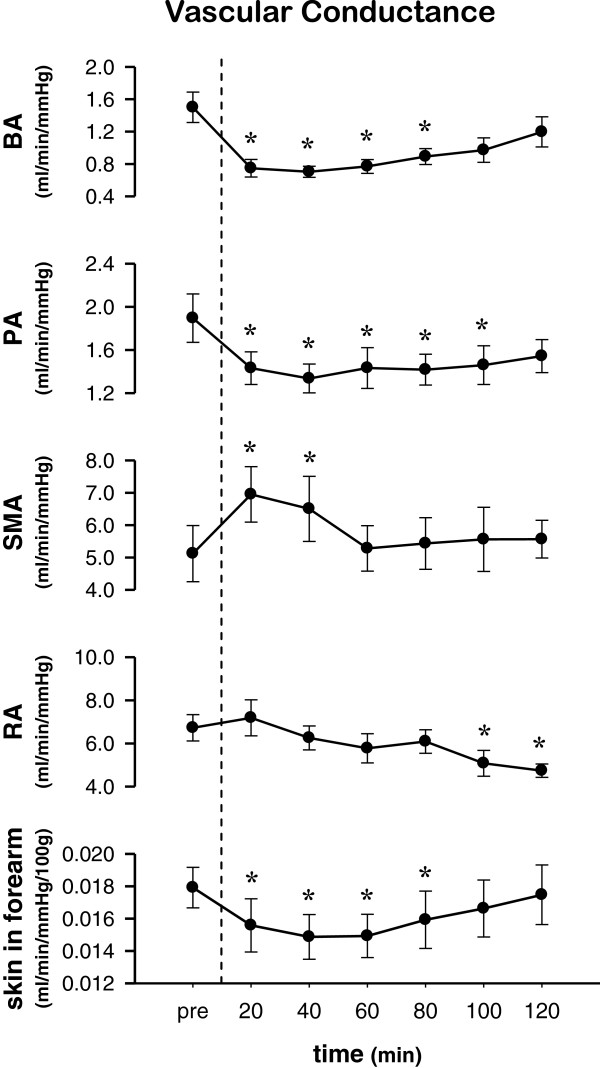
**Change in regional vascular conductance (VC) before and after ingestion of fructose.** Changes in VC in the brachial artery (BA), popliteal artery (PA), superior mesenteric artery (SMA), right renal artery (RA), and forearm skin are shown from the top. Data: mean ± SEM*: versus pre-ingestion (*P* <0.05).

VC in several peripheral regions is shown in Figure 2. VCs in SMA and RA in the rest were compatible to those which were obtained in the similar conditions in the previous studies [16,17]. VC in the BA and PA decreased rapidly after ingestion and remained decreased for approximately 90 min. In contrast, VC in the SMA increased rapidly for the first 20 to 40 min after ingestion. VC in the RA was decreased during the latter half of the protocol (that is, approximately 90 to 120 min). Finally, the VC of forearm skin decreased after ingestion and returned to baseline at approximately 90 min.

The purpose of this study was to determine acute responses of regional VC to oral fructose ingestion in young healthy humans. As expected from a previous study [7], oral fructose ingestion initiated an acute and significant elevation of MAP lasting for at least two hours. This was partially induced by a change not in TVC, but increased CO via a sustained elevation in HR. While TVC was apparently unchanged during the two hours after ingestion, there were changes in regional VC, but no net change in TVC.

The increase in BP associated with oral fructose ingestion was characterized by gradual increases in CO and HR, but no compensatory reduction in TVC (Figure 1). The acute BP elevation was partially induced by an elevation in CO via a concomitant increase in HR. This result is in agreement with previous studies [7,18]. Indirect measures using HR variability have indicated parasympathetic withdrawal and sympathetic activation compared with baseline [7]. Vollenweider *et al*. [19] showed no change in muscle sympathetic nervous activity in the lower limbs during continuous venous infusion of fructose for two hours. The increased HR in this study seemed to be induced by the parasympathetic withdraw, while the mechanism of the cardiac autonomic stimulation following ingestion of fructose remains to be established.The novel finding in the present study was that over the 120 min after fructose drink ingestion, the BP showed the acute elevation. During such duration, while the TVC was unchanged, the regional VCs in several peripheral vasculatures showed different features of changes (Figure 2). In detail, during the first 60 min after oral fructose ingestion, VC in the SMA (supply routes to the duodenum, small intestine, ascending colon, transverse colon, and pancreas) rapidly increased, whereas VC in the BA and PA (supply to mainly skeletal muscles and skin) was reduced. During the subsequent hour (that is, 60 to 120 min), VC in the SMA had already returned to baseline, whereas VC in the RA gradually decreased. In addition, VC in the BA, PA, and forearm skin gradually returned to baseline (that is, the changes in VC among the BA, PA, and forearm skin were very similar).

These results indicate that, after oral fructose ingestion, the vascular beds in peripheral limbs (that is, arms and presumably lower limbs) were vasoconstricted to compensate for vasodilation in the splanchnic region (that is, SMA) for the digestion and absorption of fructose. Such peripheral vasoconstriction seems to be derived from systemic sympathetic activation, since Jansen *et al*. [18] demonstrated acute increased serum norepinephrine levels during one hour after oral fructose ingestion.

## Conclusions/perspectives

We observed systemic and peripheral circulatory responses to oral fructose ingestion over a 2-hour period. Fructose ingestion was associated with an acute elevation in BP, which was associated with an increase in CO. Although fructose ingestion did not change TVC, there were several different changes in regional vascular responses over the period, which may, with repeated exposure, increase the risk of hypertension-induced arteriosclerosis and events such as stroke and cardiovascular disease in addition to hyperlipidemia.

## Abbreviations

BA: brachial artery; BF: blood flow; BV: blood velocity; CO: cardiac output; HR: heart rate; MAP: mean arterial blood pressure; PA: popliteal artery; RA: renal artery; SMA: superior mesenteric artery; SV: stroke volume; TVC: total vascular conductance; VC: vascular conductance.

## Competing interests

The authors declare that they have no competing interests.

## Authors’ contributions

Contribution of each author is as follows: MYE and YF conceived this study, outlined the method, directed the experiments, and interpreted the results. The majority of the article was written by MYE, CF, HK, and YF. CY and KE contributed to the experiments, data analyses, and manuscript preparation. CY, HK, and AM discussed the manuscript and aided in revising it critically for important intellectual content with MYE and YF. All authors approved the manuscript.
